# The rarer-sex effect

**DOI:** 10.1098/rstb.2021.0500

**Published:** 2023-05-08

**Authors:** Andy Gardner

**Affiliations:** School of Biology, University of St Andrews, St Andrews KY16 9TH, UK

**Keywords:** consanguinity, game theory, reproductive value, sex allocation, sex ratio, unbeatable strategy

## Abstract

The study of sex allocation—that is, the investment of resources into male versus female reproductive effort—yields among the best quantitative evidence for Darwinian adaptation, and has long enjoyed a tight and productive interplay of theoretical and empirical research. The fitness consequences of an individual's sex allocation decisions depend crucially upon the sex allocation behaviour of others and, accordingly, sex allocation is readily conceptualized in terms of an evolutionary game. Here, I investigate the historical development of understanding of a fundamental driver of the evolution of sex allocation—the rarer-sex effect—from its inception in the writing of Charles Darwin in 1871 through to its explicit framing in terms of consanguinity and reproductive value by William D. Hamilton in 1972. I show that step-wise development of theory proceeded through refinements in the conceptualization of the strategy set, the payoff function and the unbeatable strategy.

This article is part of the theme issue ‘Half a century of evolutionary games: a synthesis of theory, application and future directions’.

In some features it has an unexpectedly close similarity to certain types of situations considered in the ‘theory of games’ – W. D. Hamilton, 1967 [1. p. 477]

## 1. Introduction

The theory of games provides a toolkit for investigating rational decision making in scenarios wherein each agent's payoff depends on the decisions taken by others [2]. Its key elements are the strategy set, the payoff function and—in so-called ‘non-cooperative’ games—the concept of the Nash equilibrium [3]. The strategy set describes the options that are available to each agent, the payoff function describes how well an agent's objectives are realized as a consequence of the decisions taken by all agents, and the Nash equilibrium provides a generalization of the concept of the optimal strategy, such that when all agents adopt this strategy no agent would be able to improve their payoff by switching to a different strategy.

Evolutionary game theory is the application of this tool kit to other domains—such as the biological—in which the axiom of rationality is replaced by the action of Darwinian selection, which is understood to lead individuals to behave ‘as if’ they are rational, fitness-maximizing agents [4]. In some applications, the action of selection is implicit, and individuals are simply assumed to maximize their fitness or some proxy of this. In others, the evolutionary dynamics are explicitly described and may be of interest in their own right over and above their use in identifying equilibria and characterizing their stability. Although its boundaries with alternative evolutionary approaches—such as theoretical population genetics—are often blurred, the evolutionary game-theoretic outlook reveals itself in its use of the language of strategy.

The dawn of evolutionary game theory is typically seen as being synonymous with the publication of Maynard Smith's & Price's *The logic of animal conflict* in 1973 [5]. Yet, during the century preceding this landmark paper, the principles of evolutionary game theory had already been successfully applied to the problem of sex allocation. Here, I investigate the historical development of understanding concerning a fundamental driver of the evolution of sex allocation—the rarer-sex effect—from its inception in the writing of Charles Darwin in 1871 through to its explicit framing in terms of consanguinity and reproductive value by William D. Hamilton in 1972. I show that step-wise development of the theory proceeded through refinements in the conceptualization of the strategy set, the payoff function and the unbeatable strategy.

## 2. Charles Darwin

**Figure 1.**
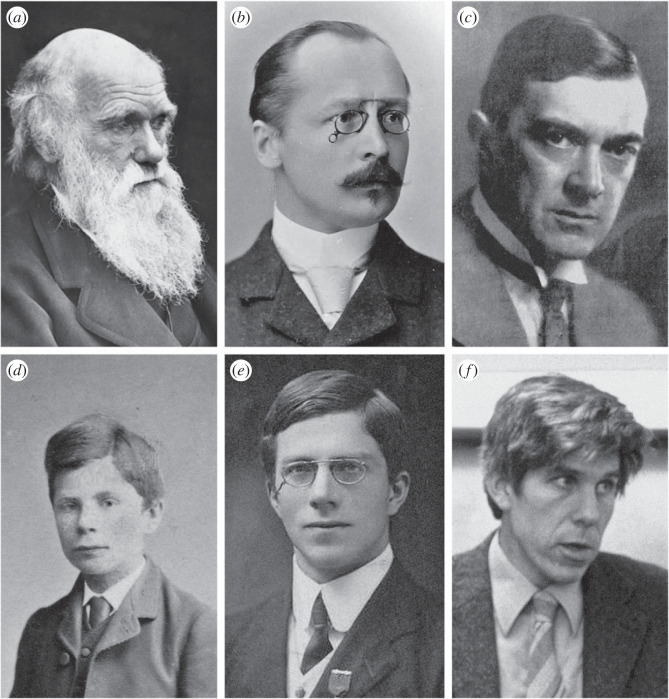
The history of the rarer-sex effect. (*a*) Charles Darwin first formulated a theory of the rarer-sex effect, focusing upon equalization of the adult sex ratio, in 1871. (*b*) Karl Gerhard Düsing provided a mathematical analysis, based on number of grandoffspring, in 1883. (*c*) Corrado Gini clarified aspects of the logic, but ultimately rejected the principle, in 1908. (*d*) John Austin Cobb (shown here aged 13–14 in 1880) reframed the theory in terms of the sex ratio of newborns, parental expenditure and reproductive value in 1914. (*e*) Ronald Aylmer Fisher provided a mathematical account of reproductive value in 1927 and 1930. (*f*) William Donald Hamilton incorporated consanguinity and developed the concept of the unbeatable strategy between 1964 and 1972. Image credits: (*a*) public domain; (*b*) Hauke Heinecke; (*c*) Italian National Institute of Statistics, [7]; (*d*) Clive Cobb; (*e*) public domain; (*f*) Sarah Hrdy.

The first person to apply Darwinian thinking to the problem of sex allocation appears to have been Charles Darwin (1809–1882 [6]; figure 1*a*) himself [8,9], in the first edition of *The descent of man* [10]. The problem, as he saw it, is to explain the approximately equal numbers of males and females among newborns in many animal populations. Earlier thinkers who had also tackled this problem include John Arbuthnot who, in 1710, argued that the unbiased sex ratio evidences divine providence in that it ensures that ‘every Male may have a Female of the same Country and suitable Age’ [11. p. 188]. Darwin's solution borrows heavily from this natural–theological outlook and simply substitutes the divine with the action of natural selection, yielding an inchoate formulation of the rarer-sex effect.

Darwin's starting point is to imagine a monogamous species in which males have—for some unspecified reason—come to outnumber females at birth, resulting in an excess of unpaired males upon these individuals reaching reproductive maturity. He supposes that, in such circumstances, if some parents have a tendency to produce fewer sons and, correspondingly, more daughters, then these parents will be more evolutionarily successful in the sense that more of their offspring would expect to reproduce, such that their heritable tendency for a relatively female-biased sex ratio will spread and act to neutralize the male bias at the population level. In his words [10, p. 316]:

Let us now take the case of a species producing from the unknown causes just alluded to, an excess of one sex—we will say of males—these being superfluous and useless, or nearly useless. Could the sexes be equalised through natural selection? We may feel sure, from all characters being variable, that certain pairs would produce a somewhat less excess of males over females than other pairs. The former, supposing the actual number of the offspring to remain constant, would necessarily produce more females, and would therefore be more productive. On the doctrine of chances a greater number of the offspring of the more productive pairs would survive; and these would inherit a tendency to procreate fewer males and more females. Thus a tendency towards the equalisation of the sexes would be brought about.

Darwin then supposes that the opposite would occur were there a female bias at the population level, such that natural selection would favour those parents who invest more into sons than daughters, which again would serve to neutralize the population-level sex-ratio bias. Moreover, he suggests that, even if parents who produce fewer offspring of the more common sex do not produce a correspondingly larger number of offspring of the rarer sex, the corrective action of natural selection may nevertheless continue to operate on account of these parents being able to allocate the same amount of reproductive resources among a smaller number of offspring and hence giving each of their rarer-sex offspring an advantage over same-sex competitors.

This formulation of the rarer-sex effect makes logical sense, but it applies only to a very specific set of circumstances. Darwin is explicit about his assumption of monogamy, but he leaves implicit an important assumption that the sex ratio among newborns is approximately equal to the sex ratio among reproductively mature adults. This is crucial because Darwin is viewing natural selection as adjusting the newborn sex ratio so as to give an adult sex ratio in which there is no excess of individuals of either sex, echoing Arbuthnot's earlier argument. Were there, for example, a strong male bias in mortality prior to reproductive maturity, then a correspondingly strong male bias among newborns would be required in order to ensure an unbiased sex ratio among adults (cf. [11]).

Darwin's logic gets into more difficulty when he attempts to relax the assumption of monogamy, as betrayed by his remark [10, p. 317]:

So it would be with polygamous species, if we assume the excess of females to be inordinately great.

That is, he implies that natural selection will not act to neutralize even a strongly female-biased sex ratio so long as all females are successfully mated. This underlines that Darwin is viewing natural selection as acting to adjust the newborn sex ratio so as to ensure an adult sex ratio in which all reproductive adults are able—in principle—to enjoy reproductive success, irrespective of the quantitative levels of reproductive success enjoyed by individual females and males in such circumstances. His account also becomes muddied by ‘for the good of the species' thinking, as he expresses a concern that the corrective action of natural selection in relation to the sex ratio might result in population growth and concomitant strain on reproductive resources, and he suggests that individuals will consequently be favoured to employ voluntary reduction of fecundity in order to ensure the continued existence of their species.

Perhaps on account of these confusions, Darwin removed his discussion of the rarer-sex effect from subsequent editions of the *The descent of man*, remarking that [12, pp. 259–260]:

I formerly thought that when a tendency to produce the two sexes in equal numbers was advantageous to the species, it would follow from natural selection, but I now see that the whole problem is so intricate that it is safer to leave its solution for the future.

Despite these difficulties, Darwin's exposition of the rarer-sex effect is notable for the sophistication with which he conceptualizes the strategy set—in particular, in his understanding that parents face a trade-off, such that the decision to invest fewer reproductive resources into offspring of one sex will naturally enable a greater investment into offspring of the other sex. His account is also remarkable in the way in which he seeks to identify the equilibrium sex ratio, not by assuming that there is a universally optimal sex ratio that maximizes the evolutionary success of parents who employ it regardless of what other parents in the population are doing, but instead by considering a range of possible population sex ratios and investigating their vulnerability to invasion by alternative sex-allocation strategies. That is, Darwin's view of the evolution of the sex ratio is fundamentally game-theoretic.

Where Darwin's argument comes unstuck—in relation to polygamy and species-level benefit—the culprit appears to be a lack of clarity concerning the appropriate payoff function. Whereas Darwin was able to reason about the adaptive evolution of most organismal traits from considerations of their consequences for lifetime reproductive success, this approach is less helpful in relation to the sex ratio, as it concerns the sex, rather than the number, of offspring. From a qualitative perspective, Darwin sees that excess, unmated individuals of the more-common sex in a monogamous species represent a waste of parental expenditure that could have instead been allocated to offspring of the rarer sex. However, from a quantitative perspective, he struggles to understand how fitness can be evaluated in polygamous species whereby, even with a strongly female-biased adult sex ratio, all females are still assured of mating success.

## 3. Karl Gerhard Düsing

Quantitative insight into the problem of the sex ratio came in the following decade, with Karl Gerhard Düsing's (1859–1924 [13]; figure 1*b*) mathematical treatment of the rarer-sex effect [9]. Düsing's work was undertaken during his doctoral studies at the University of Jena [14] and presented in several publications from 1883 to 1884. Edwards [15] provides more details of these publications—and an English-language translation of a key passage, from which I quote below.

Düsing's progress owes to his use of a concrete payoff function, which facilitates quantitative analysis. In place of the number of offspring that a mother succeeds in producing during her lifetime, which is often used as a measure of Darwinian fitness, Düsing considers her expected number of grandoffspring to provide the proper evaluation of her evolutionary success. Inviting the reader to imagine a population in which males outnumber females, he argues [15, p. 256] that:

All the males taken together have just as many offspring as all the females have (namely the same ones). Since the latter are in a minority, each of the females has on average a greater number of offspring than each of the males.

Specifically, denoting the number of females by *x*, the number of males by *nx* and the total number of offspring by *z*, Düsing calculates the expected number of offspring produced by a female as *z*/*x* and the expected number of offspring produced by a male as *z*/*nx*, such that a female can expect to produce *n* times as many offspring as a male. On that basis, he suggests that a mother who produces more daughters than sons is expected to achieve a larger number of grandoffspring than a mother who produces the same number of offspring with the opposite sex ratio, and he provides numerical examples to illustrate that a higher investment into offspring of the rarer sex—whether that be females or males—is associated with a higher expectation of grandoffspring number.

In contrast to Darwin's analysis, Düsing considers the different payoffs of individuals adopting different sex ratios not only in the context of population imbalances in the sex ratio but also in the context of populations in which there are equal numbers of males and females; in the unbiased setting he determines that a mother's expected number of grandoffspring is independent of the sex ratio of her offspring. This potentially invites the interpretation that Düsing has completed a full evolutionary game-theoretic analysis—i.e. in addition to showing that the population is expected to evolve toward an unbiased sex ratio whenever there is any sex-ratio imbalance, he has shown that this unbiased sex ratio is not evolutionarily usurped once it has been attained. However, this interpretation is too simplistic.

Düsing's framing of the rarer-sex effect is in terms of natural selection having endowed organisms with an adaptive capacity for facultative adjustment of the newborn sex ratio in response to environmentally modulated imbalances in the adult sex ratio. That is, his *x* and *nx* terms represent the total numbers of females and males of all ages, rather than at birth. His mathematical account is accompanied by a discussion of empirical observations that he suggests lend support to this hypothesis, including: data from humans and horses revealing a male bias at birth, which he appears to interpret as correcting for higher male infant mortality so that an unbiased sex ratio can be realized among adults (cf. [11]); reports of a greater number of male births in humans in the aftermath of war; and a section on arrhenotoky—the development of males from unfertilized eggs—in bees as a means of restoring sex-ratio balance when males are rare or absent. Under a literal interpretation of Düsing's hypothesis, whereby natural selection leads individuals to exclusively produce newborns of whichever sex is currently underrepresented among adults, we might expect to observe striking and sustained oscillations in sex ratio from generation to generation (cf. [1,16,17]) rather than gradual correction of sex-ratio imbalance over an evolutionary timescale.

## 4. Corrado Gini

A sharpening of the logic of the rarer-sex effect was provided by Corrado Gini (1884–1965 [18]; figure 1*c*) in his 1908 book *Il sesso dal punto di vista statistico* [9,19]. His contribution is unusual in that, while he made incisive refinements to understanding of how natural selection acts in relation to the sex ratio—in particular, clarifying the fitness payoffs associated with the production of sons versus daughters—a simple logical error ultimately led him to reject the principle.

In contrast to Darwin and Düsing, who saw the balancing of the adult sex ratio as being of central importance and the newborn sex ratio as merely an instrument by which this might be achieved, in Gini's account the selection pressures shaping sex-ratio evolution are fundamentally governed by the newborn sex ratio, and the adult sex ratio is largely irrelevant. Crucially, he argues that higher mortality of one sex prior to reproductive maturity does not lead to natural selection favouring a compensatory increase in the production of newborns of that sex in order to restore a balanced adult sex ratio, because although the surviving offspring of the higher-mortality sex have higher reproductive success a mother who produces more newborns of the higher-mortality sex has correspondingly fewer surviving offspring [9].

Gini also highlights that Darwin's and Düsing's logic implicitly assumes that the heritable factors that modulate sex ratio are passed on to—and by—both sons and daughters, such that the reproductive success of both sons and daughters contributes to a parent's evolutionary success. He argues that if, instead, sex-ratio factors were uniparentally inherited, then only offspring of the corresponding sex would count towards the evolutionary success of the corresponding parent, such that to the extent that this parent wields control over the sex ratio they would be favoured to produce only offspring of their own sex. For example, if transmission is only from mother to daughter, then mothers would be favoured to produce all-female broods. Gini makes explicit that his purpose is not to argue that sex-ratio factors are actually inherited in a uniparental manner, but rather to expose a hidden assumption of biparental inheritance that he holds to be valid. Indeed, he argues that his analysis, taken in conjunction with the empirical observation of balanced sex ratios, proves that sex-ratio factors are biparentally inherited. Nevertheless, his insights concerning the sex-ratio ramifications of uniparental inheritance—which I have not seen acknowledged elsewhere—pre-empt by several decades Lewis's [20] analysis of mitochondrially driven male sterility and Hamilton's [1] analysis of driving Y-chromosomes.

Having made these important contributions, Gini suddenly rejects the logic of the rarer-sex effect altogether, declaring that ‘Natural selection has no influence on the sex ratio at birth’ ([19, p. 328], translation mine). How does he arrive at this conclusion? Firstly, in relation to Darwin's scenario wherein mothers producing fewer offspring of the more-common sex are thereby able to produce a correspondingly larger number of offspring of the rarer sex, Gini acknowledges that such mothers will have more grandoffspring, but he argues that their impact on future generations will be exactly counterbalanced by that of less-successful but numerically superior mothers who bias the sex ratio of their offspring toward the more common sex. This argument is fallacious, as discussed by Edwards [9], in particular as it is the differential evolutionary success of mothers employing sex ratios above versus below the current population sex ratio—rather than that of mothers employing absolutely female-biased versus male-biased sex ratios—that drives sex-ratio evolution.

Secondly, in relation to the scenario wherein a mother who produces fewer offspring of the more common sex does not produce more offspring of the rarer sex but is thereby able to invest more resources into the rearing of each one of her rarer-sex offspring, providing them with an advantage over their same-sex competitors, Gini contends that Darwin's argument is one-sided and that it overlooks how a mother who produces fewer offspring of the rarer sex would thereby produce superior offspring of the more common sex. This is correct, but it does not follow that the evolutionary payoffs for investments that improve competitive ability are symmetrical with respect to sex. Indeed, a mother who improves the competitive ability of an offspring of the rarer sex would, all else being equal, expect to have a greater number of descendants than would a mother who improves the competitive ability of an offspring of the more-common sex. However, these issues would not become clear until focus had shifted away from the sex ratio and toward explicit consideration of parental expenditure in relation to each sex.

## 5. John Austin Cobb

It is in a virtually unknown paper, *The problem of the sex-ratio* by ‘J. A. Cobb’, published in 1914 in *The Eugenics Review* [21], that an understanding of the rarer-sex effect was finally reached in its more-or-less modern form, at least in relation to sexually symmetrical modes of genetic inheritance. Despite making some impact at the time, particularly upon R. A. Fisher (see below), and being highlighted in the popular periodical *The Review of Reviews* [22], this work rapidly faded into obscurity, and appears to remain there. Even following its rediscovery a quarter of a century ago by Edwards [9,23], Cobb's paper has not been cited in authoritative, book-length treatments of sex allocation theory [24,25]—or indeed, according to Web of Science and Google Scholar, by anyone else.

As no biographical information has previously been published on the author of *The problem of the sex-ratio*, it may be useful to give some details. John Austin Cobb (1866–1920; figure 1*d*) was born in Kent, England, and he attended Haileybury College, Hertfordshire, from 1879 to 1884 [26,27]. He matriculated at the University of London in 1885 but did not complete his studies [28], instead training as a solicitor and qualifying in 1889 [29]. He married in 1891 [30], and thereafter lived on private means in Minneapolis, Minnesota and then Richmond, Surrey. Although unaffiliated with any academic institution, he published a number of research articles between 1896 and 1914 in *Nature* [31,32], *Biometrika* [33] and *The Eugenics Review* [21,34,35], on topics spanning photography, statistics, the sex ratio and human fertility.

Cobb begins his account with a clear statement of the basic principle of the rarer-sex effect [21, p. 161]:

If we take the sex-ratio at birth it appears at first sight that the numbers of the sexes born will become equal. For if there are more born of one sex, say, the male, a female will have a greater chance of finding a mate than a male. There will be more matings, therefore, among the descendants of mothers of females than amongst the descendant [*sic*] of mothers of males. The mothers of females will therefore be better represented in the third generation, and as their characteristic is assumed to be inherited, there will be a tendency for the sex-ratio to diminish until it reaches equality in numbers between the sexes at birth.

He then reiterates Gini's insight that it is the sex ratio of newborns—not that of adults—that drives this action of natural selection, such that any imbalance in the adult sex ratio owing to higher mortality of one sex is irrelevant, and will not lead to adjustment of the newborn sex ratio [21, pp. 161–162]:

If we assume that males and females are conceived in equal numbers, a male at conception will have the same chance as a female of eventually finding a mate. Now if males have a higher death-rate when young the chance of mating of any male taken at random from amongst all males conceived will not be diminished. For if one male dies his chance will vanish, but the chance of the remaining males will be correspondingly increased.

Cobb then shifts attention away from the sex ratio to explicitly consider the expenditure of parental resources in relation to offspring of each sex. This provides an important refinement in conceptualization of the strategy set, and ushers in the more general problem of sex allocation as a topic for evolutionary investigation. Specifically, Cobb suggests that the slight male bias observed among newborns in humans is—after all—an evolutionary consequence of males suffering a greater incidence of childhood mortality, such that the total cost of a son is lower than that of a daughter and, accordingly, a somewhat male-biased sex ratio at birth may equalize the marginal returns from investment into offspring of each sex. In his words [21, p. 162]:

If, however, the amount of food for the family is limited a mother of boys will be able to provide for them more easily for a larger proportion of them will die early and will therefore not require so much food. This will tend to give an advantage to the brother or sister of boys over the brother or sister of girls. A brother or sister of boys will be less likely to be starved and more likely to grow to maturity and to marry. He or she will have a tendency to produce more males than a brother or sister of girls. The sex ratio will therefore rise until the less expenditure attendant upon the birth of a boy is balanced by the smaller chance a boy will have of finding a mate.

Finally, Cobb makes a crucial advance concerning the payoff function. He points out that—even for a sexually symmetrical mode of genetic inheritance—simply calculating a mother's expected number of grandchildren provides an inadequate means of evaluating her sex-allocation strategy if there is a sex difference in the average age of becoming a parent [21, pp. 162–163]:

If when the population is increasing a man selects his wife from those born two years later than himself, it gives him a larger number from whom to choose than if he took his wife from those born the same year as himself, for the number of births of girls increases from year to year. This, then, increases the man's chance of marrying and diminishes the woman's. But this biological advantage to the man is precisely balanced by the fact that the time between the birth of father and son is longer than that between mother and son. So the population does not increase so rapidly on the male side as on the female. The fact that a man marries later on the average does not therefore affect the probable number of the descendants of any male taken at random at the time of conception.

That is, in this growing-population scenario, a mother who adjusts her sex allocation in favour of sons will expect to have a larger number of grandchildren but, as these are expected to be born later than those that would have been born to her daughters, they will represent a smaller fraction of their own generation and hence contribute correspondingly less to the ancestry of future generations. And it is this asymptotic contribution to future generations—what we today term ‘reproductive value’ [36–38]—that forms the proper basis for the payoff function.

A discrepancy between the average age of mothers versus fathers leads to more of the population's reproductive value residing with the sex that reproduces at an older age [39,40], and this has implications for the action of sexually antagonistic selection [41]. However, the total reproductive value of newborn females is, as Cobb points out, equal to that of newborn males—provided that the population has attained a stable age distribution (box 1) [39]. Accordingly, sex differences in age of parentage have no biasing effect on the allocation of resources between sons versus daughters that is favoured by natural selection.

Box 1. **The mathematics of the rarer-sex effect.**

Here I show how the unbeatable sex-allocation strategy may be expressed in terms of three measures of value—marginal value, reproductive value and consanguinity—and recover the results given by Fisher [37] and Hamilton [42], respectively, for diploid and male-haploid modes of inheritance. The present account is based on that of Frank [43], who provides a fuller treatment.

Let a mother's sex-allocation strategy—i.e. the proportion of her reproductive resources that she invests into the production of sons versus daughters—be denoted by *y*, and the average sex-allocation strategy employed by mothers in the population by $\bar{y}$. Let the reproductive success of the focal mother's sons, expressed relative to the reproductive success of the average mother's sons, be denoted by *M*(*y*)/*M*($\bar{y}$), where *M* is a monotonically increasing function of its argument. Similarly, let the reproductive success of the focal mother's daughters, expressed relative to the reproductive success of the average mother's daughters, be denoted by *F*(1 − *y*)/*F*(1 − $\bar{y}$), where *F* is a monotonically increasing function of its argument. Then a small increase in the focal mother's allocation of resources to sons would serve to increase her payoff if

$$\frac{M^{\prime }(y)}{M(\bar{y})}c_{m}p_{m}>\frac{F^{\prime }(1-y)}{F(1-\bar{y})}c_{f}p_{f},$$

and a small decrease in her allocation of resources to sons would serve to increase her payoff if

$$\frac{M^{\prime }(y)}{M(\bar{y})}c_{m}p_{m}<\frac{F^{\prime }(1-y)}{F(1-\bar{y})}c_{f}p_{f},$$

where *c*_m_ is the aggregate reproductive value of all newborn males, *c*_f_ is the aggregate reproductive value of all newborn females, *p*_m_ is the consanguinity of mother and son, *p*_f_ is the consangunity of mother and daughter, and a prime denotes a derivative taken with respect to the function's argument.

Accordingly, the unbeatable sex-allocation strategy y* satisfies

$$\frac{M^{\prime }(y^{*})}{M(y^{*})}c_{m}p_{m}=\frac{F^{\prime }(1-y^{*})}{F(1-y^{*})}c_{f}p_{f}.$$

The unbeatable strategy y* is not generally independent of the shape of functions *M* and *F*. For example, if these functions take power-law form *M*(*y*) = *αy^a^* and *F*(1 − *y*) = *β*(1 − *y*)*^b^* with *a*, *b* ≤ 1 then the unbeatable sex-allocation strategy is given by

$$y^{*}=\frac{ac_{m}p_{m}}{ac_{m}p_{m}+bc_{f}p_{f}},$$

which may take any value between 0 and 1 given a suitable choice of values for *a* and *b* in scenarios in which mothers derive payoff from both sons and daughters (i.e. *c*_m_ *p*_m_, *c*_f_ *p*_f_ > 0)—see Frank [43] for more discussion. However, under certain symmetries in the *M* and *F* functions—for example, if they are both linear (i.e. *a* = *b* = 1 in the above power-law formulation) or if they have the same rate of diminishing returns (i.e. *a* = *b* < 1)—then the unbeatable sex-allocation strategy is given by

$$y^{*}=\frac{c_{m}p_{m}}{c_{m}p_{m}+c_{f}p_{f}}.$$

Under a diploid mode of inheritance, the aggregate reproductive value of newborn males is equal to that of newborn females—provided that the population has attained a stable age distribution—and the consanguinity of mother and son is equal to that of mother and daughter, and this recovers Fisher's [37] prediction of equal investment into sons and daughters,

$$y^{*}=1/2.$$

To see that the aggregate reproductive value of newborn males is equal to that of newborn females, consider a gene that is chosen from the distant future and traced back to its ancestor in the present [40]. Denote: the probability that the ancestor is male by *s*; the probability that the ancestor is female by *t* = 1 – *s*; the probability that the ancestor is newborn conditional upon being male by *u*; the probability that the ancestor is newborn conditional upon being female by *v*; and the probability that the ancestor of the gene in the previous time step was male by *s*′. The latter probability is given by *s*′ = *s* × (1/2 × *u* + 1 – *u*) + *t* × 1/2 × *v*, because if the ancestor in the present is a newborn male there is a probability 1/2 that his gene derived from his father in the previous time step and if he is not newborn then with certainty he was carrying the gene in the previous time step, and if the ancestor in the present is a newborn female then there is a probability 1/2 that her gene derived from her father in the previous time step. A stable age distribution implies *s′* = *s*, and rearranging obtains *su* = *tv*, i.e. the probability that the gene's ancestor is a newborn male is equal to the probability that it is a newborn female.

Owing to the sexual symmetry of diploid inheritance it is intuitive that the consanguinity of mother and son is equal to that of mother and daughter. The consanguinity of mother and offspring—i.e. the probability that a gene drawn from a particular locus from the mother and a gene drawn from the same locus from the offspring are identical by descent—is given by 1/2 × (1/2 + 1/2 × *f*) + 1/2 × *f* = (1 + 3*f*)/4, where *f* is the consanguinity of mating partners, and irrespective of the sex of the offspring [44].

Under a male-haploid mode of inheritance, the aggregate reproductive value of newborn females is twice that of newborn males—provided that the population has attained a stable age distribution. To see this, note that repeating the same procedure as above obtains *s*′ = *s* × (1 – *u*) + *t* × 1/2 × *v* under male haploidy, as newborn males receive all of their genes from their mothers, and setting *s*′ = *s* and rearranging obtains *tv* = 2*su* [40]. Moreover, the consanguinity of mother and son may differ from that of mother and daughter: for daughters, this is (1 + 3*f*)/4 as before; but for sons it is 1/2 × 1 + 1/2 × *f* = (1 + *f*)/2. Making these substitutions yields Hamilton's [42] prediction for the unbeatable sex-allocation strategy,

$$y^{*}=\frac{1+f}{2+4f}.$$

In an outbred population (*f* = 0) the unbeatable sex-allocation strategy under male haploidy is to invest equally into sons and daughters, because although newborn females have twice the reproductive value of newborn males, the consanguinity of mother and son is twice that of mother and daughter, leading to no overall bias in valuation. In a chronically inbred population (*f* = 1) the unbeatable sex-allocation strategy is to invest twice as much into daughters as sons, as here mothers are equally consanguinous with son and daughter, and hence the higher reproductive value of newborn females favours twice as much investment into daughters as into sons. Note that inbreeding will often be associated with additional fitness consequences—such as local competition for mates—that have not been considered here but are also expected to modulate the unbeatable sex-allocation strategy [1].

## 6. Ronald Aylmer Fisher

Cobb's work had an enormous influence on Ronald Aylmer Fisher (1890–1962 [45]; figure 1*e*), who is traditionally—though incorrectly—credited as the originator of the theory of the rarer-sex effect [46,47]. Fisher is explicit in crediting Cobb's 1913 paper on *Human fertility* [34] as providing the inspiration for his own theory of the decline of civilizations, articulated in the latter chapters of his 1930 book *The genetical theory of natural selection* [37]. And although he does not explicitly credit Cobb for the sex-ratio argument that he gives earlier in the same book, it is beyond reasonable doubt that Fisher was aware of Cobb's sex-ratio paper. Edwards [9] compiles convincing circumstantial evidence, including, for example, the fact that Cobb's paper appeared in the same journal issue as two book reviews contributed by Fisher [48,49].

Fisher's major contribution to understanding of the rarer-sex effect is to give it a more formal foundation by providing a proper mathematical account of—and name for—the concept of reproductive value. This is done firstly in an article on *The actuarial treatment of official birth records* published in 1927 in *The Eugenics Review* [36], and also in *The genetical theory of natural selection* [37, pp. 27–30], again without making any reference to Cobb's work. Fisher then gives what is essentially Cobb's formulation of the rarer-sex effect—with one crucial difference [37, pp. 142–143]:

Let us consider the reproductive value of these offspring at the moment when this parental expenditure on their behalf has just ceased. If we consider the aggregate of an entire generation of such offspring it is clear that the total reproductive value of the males in this group is exactly equal to the total value of all the females, because each sex must supply half the ancestry of all future generations of the species. From this it follows that the sex ratio will so adjust itself, under the influence of Natural Selection, that the total parental expenditure incurred in respect of children of each sex, shall be equal; for if this were not so and the total expenditure incurred in producing males, for instance, were less than the total expenditure incurred in producing females, then since the total reproductive value of the males is equal to that of the females, it would follow that those parents, the innate tendencies of which caused them to produce males in excess, would, for the same expenditure, produce a greater amount of reproductive value; and in consequence would be the progenitors of a larger fraction of future generations than would parents having a congenital bias towards the production of females. Selection would thus raise the sex-ratio until the expenditure upon males became equal to that upon females.

Fisher's key innovation here is to specify that the parental expenditure upon each sex will be equalized by natural selection. Cobb had made no such claim and, indeed, it is not generally true. The equal-expenditure result hinges upon the implicit assumption that when total parental expenditure upon each sex is equal then a further small investment into sons would yield the same share of their class's total reproductive value as would the same small investment into daughters—such that the equality of the class reproductive values ensures that the overall returns to the parent are the same irrespective of into which sex the investment is made (box 1) [43]. But this need not be the case.

For example, it might be that a son's expected mating success exhibits such strongly diminishing returns on parental expenditure that a further increment of investment would not appreciably improve his reproductive success, whereas the same additional investment into a daughter might meaningfully increase her fecundity, making the latter a better investment even if the overall sex allocation of the population is already somewhat female-biased. Such complexities are more likely to arise in taxa exhibiting smaller broods and extended parental care—such as birds and mammals—whereby adjustments to sex allocation are more likely to involve differences in provisioning rather than an alteration of sex ratio, and are more reasonably neglected in taxa in which a mother's numerous offspring each receive standard provisionment and are scattered shortly after conception—as for example in many insects—whereby the returns on investment into each sex might be more-or-less linear [50].

## 7. William Donald Hamilton

The final fundamental contributions to understanding of the rarer-sex effect were made by William Donald Hamilton (1936–2000 [51]; figure 1*f*), who showed how consanguinity enters into the payoff function and developed the concept of the ‘unbeatable’ strategy. As discussed above, Gini had pointed out that previous treatments of the rarer-sex effect implicitly assumed biparental inheritance such that there is no fundamental asymmetry in the value of sons and daughters in the absence of a population-level bias in sex allocation, and had worked out the sex-ratio consequences of uniparental inheritance that leads a parent to value only offspring of the corresponding sex. But beyond these qualitative extremes, it was unclear how the logic of the rarer-sex effect would play out under sexually asymmetrical modes of inheritance—such as male haploidy, as exhibited by ants, bees and wasps—in which sons and daughters should both appear within a mother's payoff function but in a potentially asymmetrical way. And although from its inception in the work of Darwin the rarer-sex effect had a game-theoretic flavour, Hamilton was the first to explicitly make this point.

Hamilton's contributions to sex-allocation theory grew out of his more basic work on inclusive fitness, in which he showed that natural selection is expected to lead organisms to appear as if they are maximizing the overall reproductive success of their relatives—including themselves—with each increment or decrement being weighted by the degree of consanguinity between the actor and corresponding recipient [52]. Here, consanguinity refers to the probability that genes drawn from two parties—from the same locus—are identical by descent; the ratio of the consanguinity of actor and recipient to the consanguinity of the actor to herself defines the well-known coefficient of relatedness [44]. Hamilton's first remarks on the evolution of the sex ratio appear in part II of his 1964 paper *The genetical evolution of social behaviour* [53], and here he seems to correctly grasp the solution in outline, namely that a mother should weight each of her newborns according to her consanguinity to each and also their reproductive values, these two measures of value combining in a multiplicative way (box 1). However, in his first attempt to calculate these quantities as they apply to male-haploid species, he appears to make two errors, and this leads him to derive the unusual prediction of a sex ratio in which females outnumber males by a factor *ϕ* = (1 + √5)/2 ≈ 1.618—that is, the ‘golden ratio’, which has been celebrated for its aesthetically pleasing properties since antiquity.

Hamilton's first error concerns his calculation of consanguinity [54]. In an outbred, male-haploid population the consanguinity of mother and son is double that of mother and daughter because whereas males develop from unfertilized eggs and thereby derive all of their genes from their mothers, females develop from fertilized eggs and thereby derive half of their genes from each parent in the usual way (box 1). However, Hamilton [53] employs a faulty method for calculating consanguinity, which involves assigning haploid males a second ‘cipher’ gene at each locus, so that he can treat them as if they are diploid, and this leads him to calculate the consanguinity of mother and son at half of its true value.

This consanguinity error alone does not explain how Hamilton arrives at a sex ratio in proportion to the golden ratio, so it appears that he also makes a further error, in his calculation of reproductive value. In male-haploid species, the probability that a gene drawn from a distant future generation traces its origin back to a newborn female in the present generation is twice the probability that it traces back to a newborn male, independently of the sex ratio, and hence the aggregate reproductive value of the newborn females is twice that of the newborn males (box 1) [40]. Yet, had Hamilton used these correct reproductive values with the incorrect consanguinities he would have arrived at a sex ratio in which females outnumber males 2 to 1. In order to recover a sex ratio of *ϕ* females for every male, it appears that Hamilton is calculating the reproductive value of the females as being *ϕ* times that of the males.

It is unclear how Hamilton obtains these erroneous reproductive values. One possibility is that he is attempting to calculate the asymptotic proportions of females and males in a genealogy within which each female descendant gives rise to a single son and a single daughter and each male descendant gives rise to a single daughter. In each successive generation the number of females is given by consecutive Fibonacci numbers, with the number of males trailing one Fibonacci number behind, and so in the long run their ratio converges upon *ϕ* [55]. Whatever the reason for the appearance of *ϕ*, Hamilton's sex-ratio prediction is repeated in the bestselling 2003 novel *The da Vinci Code* [56, ch. 20], and must therefore be in the running for the most widely read—albeit incorrect—quantitative result in the history of evolutionary biology:

‘Ever study the relationship between females and males in a honeybee community?’

‘Sure. The female bees always outnumber the male bees.’

‘Correct. And did you know that if you divide the number of female bees by the number of male bees in any beehive in the world, you always get the same number?’

‘You do?’

*‘Yup. PHI.’*

The girl gaped. ‘NO WAY!’.

In 1971, Hamilton [54] points out his error concerning the calculation of consanguinity—though not his error concerning the calculation of reproductive value, which he appears to silently correct—and remarks that mothers in male-haploid species are in fact favoured to employ an unbiased sex ratio because, although at this equilibrium point daughters have twice the reproductive value of sons, the consanguinity of mother and son is twice that of mother and daughter. In 1972, he shows his working and generalizes the result to allow for inbreeding, which increases the consanguinity of mother and daughter relative to the consanguinity of mother and son, and hence favours a somewhat female-biased sex ratio (box 1) [42]. By this time, he has also shown that inbreeding life-histories can have consequences not only for consanguinity of daughters and sons but also for fitness interactions between collateral relatives, yielding a further selection pressure—termed ‘local mate competition’—that moulds sex allocation in addition to the basic rarer-sex effect [1].

In addition to establishing the role of consanguinity, Hamilton also makes the first explicit link between the rarer-sex effect and the theory of games, and develops the concept of the ‘unbeatable’ strategy as an evolutionary biology analogue of the Nash equilibrium, in his 1967 paper *Extraordinary sex ratios* [1]. Here he is building upon the work of Verner [57], who—in 1965—had first applied the term ‘strategy’ to sex-ratio evolution, and MacArthur [58] who—also in 1965—had, in effect, first calculated the sex-ratio that was the best response to itself, but struggled to articulate exactly what this represented. In particular, Hamilton points out that the rarer-sex effect has a ‘game-like feature … in the sense of a play by the individual against the population’ and that the resemblance to the ‘theory of games … becomes accentuated as we proceed into circumstances of local competition’ that take the form of the *n*-player games studied by game theorists [1, p. 477].

And he analyses these various sex-ratio scenarios in an explicitly game-theoretic manner, using differential calculus (box 1). First, he mathematically describes the payoff—i.e. relative contribution of ancestry to future generations—for an individual who adopts a variant sex-ratio strategy in the context of a population in which every other individual adopts a given resident strategy. Second, he uses this payoff function to determine whether variants with sex ratios above or below the resident strategy enjoy a greater payoff than does the resident itself, such that natural selection would act to favour these variants over the resident strategy. And, third, he uses this information to determine which resident strategy cannot be beaten by any variant in this way, yielding the unbeatable sex-ratio strategy.

## 8. Epilogue

The foregoing account of the historical development of understanding of the rarer-sex effect differs from some others that have been given elsewhere. Traditionally, Fisher has been identified as the originator of the rarer-sex principle [46,47], though in the last quarter century there has been growing acknowledgement that he was drawing upon the work of others. Edwards [9] has highlighted the contributions of Darwin and Düsing, though somewhat overstating the extent to which their reasoning—which hinges upon the adult sex ratio—lines up with modern understanding, and he has drawn attention to the work of Gini and Cobb, though without commenting upon some important aspects of their respective contributions—such as the consequences of uniparental inheritance and the fundamental importance of reproductive value. Hamilton has been celebrated for his work on extraordinary sex ratios, yet this has tended to overshadow his contribution to the understanding of the unbiased sex ratio, particularly in relation to male haploidy. Finally, I have passed over, without comment, a number of celebrated works that do not appear to have actually advanced understanding, such as Shaw & Mohler's 1953 paper *The selective significance of the sex ratio* [59], of which the supposed innovation to calculate expected number of grandoffspring had already been made 70 years earlier by Düsing (cf. [9]) and superseded 39 years earlier by Cobb (though it did help to bring Fisher's account of the rarer-sex effect to wider attention).

My account has made clear that game-theoretic thinking was being productively applied within evolutionary biology throughout the century preceding the 1973 publication of Maynard Smith and Price's *The logic of animal conflict* [5]. Indeed, the game-theoretic treatment of the rarer-sex effect was itself a crucial motivator for Maynard Smith and Price's investigation of ‘evolutionarily stable’ strategies, as is apparent not only from their explicit acknowledgement of MacArthur's and Hamilton's sex-ratio work but also from G. R. Price's interchangeable use of the terms ‘unbeatable’ and ‘stable against evolutionary perturbation’ in his unpublished 1968 manuscript *Antlers, intraspecific combat, and altruism* [60], which was to form the basis of his later publication with Maynard Smith.

Following the elucidation of the fundamental driver of selection in relation to sex allocation—the rarer-sex effect—and the incorporation of additional selective drivers, such as local mate competition, which greatly expanded the scope for empirical testing of comparative predictions, sex-allocation has flourished as a topic of evolutionary investigation [24,25]. This owes in large part to a tight interplay of theoretical and empirical research, which has been facilitated by the embrace of strategic, game-theoretic approaches [43,61,62] that dispense with population-specific—and largely unknowable—genotypic details in favour of economic analysis of life-history decisions that can be readily compared and contrasted between populations and taxa. Moreover, the relative ease of its measurement, the relatively well-understood nature of its central trade-off and the relatively strong connection that it has with differential evolutionary success has meant that the sex ratio has yielded arguably the best quantitative, empirical support for Darwinian adaptation [25].

## Data Availability

This article has no additional data.

## References

[1] Hamilton WD. 1967 Extraordinary sex ratios. Science **156**, 477-488. (10.1126/science.156.3774.477) 6021675

[2] von Neumann J, Morgenstern O. 1944 Theory of games and economic behavior. Princeton, NJ: Princeton University Press.

[3] Nash JF. 1950 Equilibrium points in *n*-person games. Proc. Natl Acad. Sci. USA **36**, 48-49. (10.1073/pnas.36.1.48) 16588946 PMC1063129

[4] Maynard Smith J. 1982 Evolution and the theory of games. Cambridge, UK: Cambridge University Press.

[5] Maynard Smith J, Price GR. 1973 The logic of animal conflict. Nature **246**, 15-18. (10.1038/246015a0)

[6] Browne J. 1995 Charles Darwin: voyaging. London, UK: Jonathan Cape.

[7] Leti G. 1996 *L'istat e il consiglio superiore di statistica dal 1926 al 1945* [*Istat and the high council of statistics from 1926 to 1945*]. Rome, Italy: Istituto Nazionale di Statistica. [In Italian.]

[8] Sober E. 1984 The nature of selection. Chicago, IL: University of Chicago Press.

[9] Edwards AWF. 1998 Natural selection and the sex ratio: Fisher's sources. Am. Nat. **151**, 564-569. (10.1086/286141) 18811377

[10] Darwin CR. 1871 The descent of man, and selection in relation to sex, 1st edn. London, UK: John Murray.

[11] Arbuthnot J. 1710 An argument for divine providence, taken from the constant regularity observ'd in the births of both sexes. Phil. Trans. R. Soc. Lond. **27**, 186-190. (10.1098/rstl.1710.0011)

[12] Darwin CR. 1874 The descent of man, and selection in relation to sex, 2nd edn. London, UK: John Murray.

[13] Heinecke U. 2002 Düsing, Karl Gerhard. In Magdeburger biographisches lexikon [*Magdeburg biographical lexicon*] (eds G Heinrich, G Schandera), pp. 146-147. Magdeburg, Germany: Scriptum Verlag Magdeburg. [In German.]

[14] Düsing K. 1883 Die factoren, welche die Sexualität entscheiden [The factors that decide sexuality]. PhD dissertation, University of Jena, Gustav Fischer. [In German.]

[15] Edwards AWF. 2000 Carl Düsing (1884) on *The regulation of the sex ratio*. Theor. Popul. Biol. **58**, 255-257. (10.1006/tpbi.2000.1482) 11120652

[16] Williams CB. 1917 Some problems of sex ratios and parthenogenesis. J. Genet. **6**, 255-267. (10.1007/BF02981871)

[17] Gardner A. 2014 Dynamics of sex ratio and female unmatedness under haplodiploidy. Ecol. Evol. **4**, 1623-1628. (10.1002/ece3.1045) 24967080 PMC4063463

[18] Castellano V. 1965 Corrado Gini: a memoir. Metron **24**, 2-84.

[19] Gini C. 1908 *Il sesso dal punto di vista statistico* [*Sex from a statistical point of view*]. Milan, Italy: R. Sandron. [In Italian.]

[20] Lewis D. 1941 Male sterility in natural populations of hermaphrodite plants. The equilibrium between females and hermaphrodites to be expected with different types of inheritance. New Phytol. **40**, 56-63. (10.1111/j.1469-8137.1941.tb07028.x)

[21] Cobb JA. 1914 The problem of the sex-ratio. Eugen. Rev. **6**, 157-163. 21259588 PMC2986874

[22] Cobb JA. 1914 Sex ratio. *Rev. Rev.* **50**, 128. PMC2986874 21259588

[23] Edwards AWF. 1997 The Galton Lecture: The Eugenics Society and the development of biometry. In Essays in the history of eugenics (ed. RA Peel), pp. 156-172. London. UK: Galton Institute.

[24] Hardy ICW. 2002 Sex ratios – concepts and research methods. Cambridge, UK: Cambridge University Press.

[25] West SA. 2009 Sex allocation. Princeton, NJ: Princeton University Press.

[26] Milford LS. 1891 Haileybury Register, 1862–1891. Hertford, UK: Stephen Austin & Sons.

[27] Deaths. *Times,* 2 November 1920, p. 1.

[28] University of London. 1899 *University of London General Register Part II*, p. 33.

[29] Law students’ journal—results at the Easter examinations. *Solicit. J.*, 25 May 1889, p. 473.

[30] Society’s giddy whirl. *Minneapolis Tribune*, 13 September 1891, p. 10.

[31] Cobb JA. 1896 Measurement of crabs. Nature **55**, 155. (10.1038/055155b0)

[32] Cobb JA. 1905 Halation. Nature **73**, 54. (10.1038/073054c0)

[33] Cobb JA. 1908 The effect of errors of observation upon the correlation coefficient. Biometrika **6**, 109. (10.2307/2331561)

[34] Cobb JA. 1913 Human fertility. Eugen. Rev. **4**, 379-382. 21259544 PMC2986900

[35] Cobb JA. 1914 The alleged inferiority of the first-born. Eugen. Rev. **5**, 357-359. 21259580 PMC2987001

[36] Fisher RA. 1927 The actuarial treatment of official birth records. Eugen. Rev. **19**, 103-108. 21259852 PMC2987494

[37] Fisher RA. 1930 The genetical theory of natural selection. Oxford, UK: Clarendon Press.

[38] Grafen A. 2006 A theory of Fisher's reproductive value. J. Math. Biol. **53**, 15-60. (10.1007/s00285-006-0376-4) 16791649

[39] Grafen A. 2014 Total reproductive value for females and for males in sexual diploids are not equal. J. Theor. Biol. **359**, 233-235. (10.1016/j.jtbi.2014.05.021) 24859413

[40] Gardner A. 2014 Total reproductive value of juvenile females is twice that of juvenile males under X-linkage and haplodiploidy. J. Theor. Biol. **359**, 236-237. (10.1016/j.jtbi.2014.06.036) 25017725

[41] Hitchcock TJ, Gardner A. 2020 A gene's-eye view of sexual antagonism. Proc. R. Soc. B **287**, 20201633. (10.1098/rspb.2020.1633) PMC7575522 32781951

[42] Hamilton WD. 1972 Altruism and related phenomena, mainly in social insects. Annu. Rev. Ecol. Syst. **3**, 193-232. (10.1146/annurev.es.03.110172.001205)

[43] Frank SA. 1998 Foundations of social evolution. Princeton, NJ: Princeton University Press.

[44] Bulmer M. 1994 Theoretical evolutionary ecology. Sunderland, MA: Sinauer Associates.

[45] Fisher Box J. 1978 R. A. Fisher: the life of a scientist. Baltimore, MD: John Wiley & Sons.

[46] Crow JF, Kimura M. 1970 An introduction to population genetics theory. Manhattan, NY: Harper & Row.

[47] Dawkins R. 1976 The selfish gene. Oxford, UK: Oxford University Press.

[48] Fisher RA. 1914 Mechanism, life, and personality: an examination of the mechanistic theory of life and mind. Eugen. Rev. **6**, 165.

[49] Fisher RA. 1914 The family in its sociological aspects. Eugen. Rev. **6**, 165-166.

[50] Frank SA. 1990 Sex allocation theory for birds and mammals. Annu. Rev. Ecol. Syst. **21**, 13-55. (10.1146/annurev.es.21.110190.000305)

[51] Segerstrale U. 2013 Nature's oracle: the life and work of W. D. *Hamilton*. Oxford, UK: Oxford University Press.

[52] Hamilton WD. 1964 The genetical evolution of social behaviour. I. J. Theor. Biol. **7**, 1-16. (10.1016/0022-5193(64)90038-4) 5875341

[53] Hamilton WD. 1964 The genetical evolution of social behaviour. II. J. Theor. Biol. **7**, 17-52. (10.1016/0022-5193(64)90039-6) 5875340

[54] Hamilton WD. 1971 The genetical evolution of social behavior. II. In Group selection (ed. GC Williams), pp. 44-89. Piscataway, NJ: Transaction Publishers.

[55] Yanega D. 1996 Sex ratio and sex allocation in sweat bees (Hymenoptera: Halictidae). J. Kansas Entomol. Soc. **69**, 98-115.

[56] Brown D. 2003 The da Vinci code. New York, NY: Doubleday.

[57] Verner J. 1965 Selection for sex ratio. Am. Nat. **99**, 419-421. (10.1086/282384)

[58] MacArthur RH. 1965 Ecological consequences of natural selection. In Theoretical and mathematical biology (eds TH Waterman, HJ Morowitz), pp. 388-397. New York, NY: Blaisdell Publishing Company.

[59] Shaw RF, Mohler JD. 1953 The selective significance of the sex ratio. Am. Nat. **87**, 337-342. (10.1086/281794)

[60] Price GR. 1968 *Antlers, intraspecific combat and altruism*. George Price Papers, British Library, London.

[61] Charnov EL. 1982 The theory of sex allocation. Princeton, NJ: Princeton University Press.

[62] Taylor PD, Frank SA. 1996 How to make a kin selection model. J. Theor. Biol. **180**, 27-37. (10.1006/jtbi.1996.0075) 8763356

